# Prevention and Management of Recurrent Laryngeal Nerve Palsy in Minimally Invasive Esophagectomy: Current Status and Future Perspectives

**DOI:** 10.3390/jcm13247611

**Published:** 2024-12-13

**Authors:** Yusuke Taniyama, Hiroshi Okamoto, Chiaki Sato, Yohei Ozawa, Hirotaka Ishida, Michiaki Unno, Takashi Kamei

**Affiliations:** Department of Surgery, Tohoku University Graduate School of Medicine, 2-1 Seiryo-machi, Aoba-ku, Sendai 980-8575, Miyagi, Japan

**Keywords:** esophageal cancer, minimally invasive esophagectomy, recurrent laryngeal nerve

## Abstract

Recurrent laryngeal nerve palsy remains a significant complication following minimally invasive esophagectomy for esophageal cancer. Despite advancements in surgical techniques and lymphadenectomy precision, the incidence of recurrent laryngeal nerve palsy has not been improved. Recurrent laryngeal nerve palsy predominantly affects the left side and may lead to unilateral or bilateral vocal cord paralysis, resulting in hoarseness, dysphagia, and an increased risk of aspiration pneumonia. While most cases of recurrent laryngeal nerve palsy are temporary and resolve within 6 to 12 months, some patients may experience permanent nerve dysfunction, severely impacting their quality of life. Prevention strategies, such as nerve integrity monitoring, robotic-assisted minimally invasive esophagectomy, and advanced dissection techniques, aim to minimize nerve injury, though their effectiveness varies. The management of recurrent laryngeal nerve palsy includes voice and swallowing rehabilitation, reinnervation techniques, and, in severe cases, surgical interventions such as thyroplasty and intracordal injection. As recurrent laryngeal nerve palsy can lead to significant postoperative respiratory complications, a multidisciplinary approach involving surgical precision, early detection, and comprehensive rehabilitation is crucial to improving patient outcomes and minimizing long-term morbidity in minimally invasive esophagectomy. This review article aims to inform esophageal surgeons and other clinicians about strategies for the prevention and management of recurrent laryngeal nerve palsy in esophagectomy.

## 1. Introduction

Lymph node dissection around the recurrent laryngeal nerve (RLN) is a critical component of esophageal cancer surgery, especially in thoracic esophageal carcinoma, where metastasis to the lymph nodes around RLN is frequently observed. The incidence of this RLN lymph node metastasis in thoracic esophageal cancer is reported to be between 30% and 50% [1,2,3,4]. Although RLN lymph node metastasis is associated with poorer overall survival compared to cases without nodal involvement [3,4,5], comprehensive RLN lymphadenectomy has been shown to improve survival outcomes and reduce the risk of local recurrence [6,7]. However, this procedure is technically challenging due to the close proximity of the RLN, increasing the risk of nerve injury, which can lead to vocal cord paralysis and aspiration.

Recent advances in minimally invasive surgery for esophageal cancer (MIE) have improved the precision of lymphadenectomy, including the dissection of RLN lymph nodes, as evidenced by a study showing a significant increase in the number of RLN lymph nodes retrieved compared to that via traditional open approaches [8,9,10]. Also, the enhanced visualization provided by thoracoscopic techniques allows for more precise dissection while reducing tissue trauma around the RLN, minimizing the risk of RLN palsy. However, despite these advancements in surgical precision, the incidence of postoperative RLN palsy has not significantly decreased between minimally invasive and open surgical approaches, suggesting that while MIE improves lymphadenectomy accuracy, it does not necessarily mitigate the risk of nerve damage [9,10,11]. This can be attributed to several factors. First, the complexity of lymph node dissection around the RLN remains challenging, regardless of the surgical approach. Second, the prolonged operative time of MIE may increase the risk of RLN injury. Furthermore, the improved visualization provided by MIE, which allows for better identification of the RLN, may encourage more aggressive lymph node dissection in this region compared to open thoracotomy, inadvertently raising the risk of nerve damage. As a result, RLN palsy rates remain similar between MIE and open procedures, emphasizing the need for refined surgical techniques and post-operative management to reduce RLN palsy and fatal complication. Given those considerations, this review will address the ongoing issue of RLN palsy in MIE, discussing current strategies and management for improving surgical outcomes and patient quality of life.

## 2. Risk

Reported incidences of RLN palsy in MIE vary widely, with several studies documenting rates between 11% and 88%, with variations depending on the type of surgery, the definition of RLN palsy, and the patient population [9,10,12,13,14]. Although it usually occurs unilaterally, mostly on the left side, 1.7–15.9% of cases were bilateral [1,12,14,15]. The left RLN is longer compared to the right side, which requires a longer dissection distance, making it more prone to palsy. Additionally, since the approach to the thoracic cavity is usually from the right side, the esophagus tends to be retracted forward during dissection, causing significant flexion of the RLN via the esophageal branches, which can be one of the reasons for the RLN palsy. The McKeown procedure, which involves a three-field lymphadenectomy, is often associated with a higher risk of RLN injury due to extensive lymph node dissection around the RLN, while Ivor Lewis has a lower incidence of RLN palsy [16,17]. This can be attributed not only to the omission of cervical lymph node dissection around the RLN lymph node but also to the fact that aggressive lymph node dissection around the RLN is generally not required in the patient for which this Ivor Lewis procedure is indicated. RLN palsy is also influenced by patient-specific anatomical factors. According to a study published, a narrower RLN is more susceptible to injury due to reduced resistance to traction and compression, as it offers less resistance to traction and compression [18]. Additionally, preoperative CT imaging reveals RLN anatomical variations and proximity to critical surgical areas, aiding in risk assessment and surgical planning [19].

The areas of upper mediastinal LN dissection are anatomically close to the RLN, and the presence of large tumor or lymph node metastasis in this area increases the risk of direct nerve trauma, traction injury, or thermal damage from electrocautery. The method of dissection, such as blunt dissection, influences the risk of nerve damage due to stretching or compression [20]. Thermal damage is another significant risk, as the heat generated during hemostasis or tissue dissection can inadvertently affect the RLN, leading to nerve dysfunction [21]. Especially under wet conditions, the high temperature of steam from the energy device can easily cause thermal damage, and steam-safe distances of 3 mm for ultrasonic devices and 10 mm for bipolar sealing devices are recommended [21].

Most of the cases of RLN palsy result in temporary impairment which would recover within 6–12 months [22,23]. However, the recovery process of the recurrent laryngeal nerve (RLN) is highly complex and depends on the severity of the injury. Some patients with Sunderland class II or III RLN injuries, where the nerve remains structurally intact but sustains significant damage, including Wallerian degeneration, may experience permanent RLN palsy. In such cases, the injured RLN may partially reinnervate the laryngeal muscles, leading to synkinesis, where abductor fibers innervate adductor muscles and vice versa. Eventually, the affected vocal fold remains immobile in an adducted position [24]. Permanent RLN palsy occurs in approximately 4–13% of cases following esophagectomy [12,25], particularly when aggressive dissection is required near the nerve’s pathway [26]. If symptoms persist beyond six months to one year, the likelihood of complete recovery diminishes significantly [22,23].

## 3. Influence

In the early postoperative period, RLN palsy can lead to vocal cord palsy, resulting in hoarseness, dysphagia, and an impaired ability to cough effectively when it happens unilaterally [12,14,25,26]. On the other hand, bilateral vocal cord palsy would cause airway obstruction, dyspnea, and stridor, which could be an emergency situation and potentially require tracheostomy [14,15]. Hoarseness is one of the most common symptoms of unilateral RLN palsy which affects the movement of the vocal cord and impacts the patient’s ability to communicate. However, some cases do not cause hoarseness, even if RLN palsy has occurred. This is because the vocal cord on the unaffected side would compensate by moving towards the affected side, allowing for glottic closure and vocalization. Therefore, RLN palsy cannot be accurately diagnosed just by the presence of hoarseness alone and needs laryngoscopy for an exact diagnosis. RLN palsy also causes limited inversion of the epiglottis by the impairment of the elevating hyoid bone [27] and results in aspiration, dysphagia, and ineffective cough. Dysphagia after esophagectomy makes oral intake difficult due to the incomplete closure of the glottis and weakened swallowing function, leading to a poor quantity of food intake and worsening quality of life [28]. Ineffective cough results in reduced intrathoracic pressure generation necessary for an effective cough reflex. This inability to close glottis can also cause the impairment of airway protection, which may lead to aspiration and further lead to pneumonia. This aspiration pneumonia is one of the most common and serious postoperative complications caused by RLN palsy, prolonging hospital stays and increasing morbidity [12,29,30,31]. Of the patients with RLN palsy, 22% had postoperative pneumonia, compared with 10% of those without palsy [12]. On the other hand, RLN palsy itself does not necessarily contribute to pneumonia-related mortality or overall survival [12,31,32,33]. This indicates that RLN palsy would induce respiratory complication in the short term but may not have a significant impact on long-term prognosis. However, adjuvant therapies, including checkpoint inhibitors, have been increasingly employed in the treatment of esophageal cancer [34]. Delays in initiating adjuvant therapy due to complications such as pneumonia caused by RLN palsy may potentially impact patient prognosis. Although further investigation is warranted, ensuring a seamless transition to adjuvant therapy for postoperative patients remains a critical consideration.

## 4. Prevention

**Figure 1:**
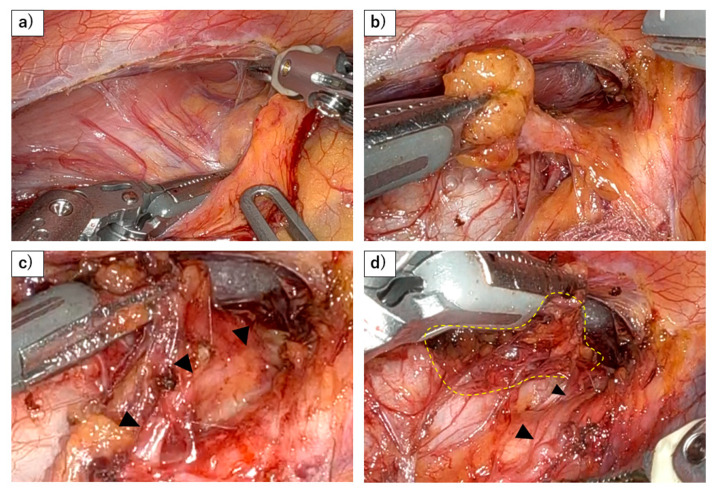
Dissection of right RLN lymph nodes using a robot. (**a**) Dorsal side of lymph nodes along the right recurrent laryngeal nerve (RLN) are being dissected as the same plane with the dorsal side of the esophagus. This figure demonstrates that the lymphatic chain forming these nodes exhibits a mesenteric-like structure. (**b**) The lymphatic chain is dissected from the trachea, with the right recurrent laryngeal nerve and subclavian artery serving as the base, resembling a mesenteric structure. (**c**) As the lymphatic chain is dissected dorsally from the right subclavian artery, the recurrent laryngeal nerve (black arrowhead) naturally becomes visible under the thin membrane. (**d**) After dividing the esophageal branch of the recurrent laryngeal nerve and dissecting from the lateral wall of the trachea, en bloc resection of the lymphatic chain will be possible (yellow dots).

**Figure 2:**
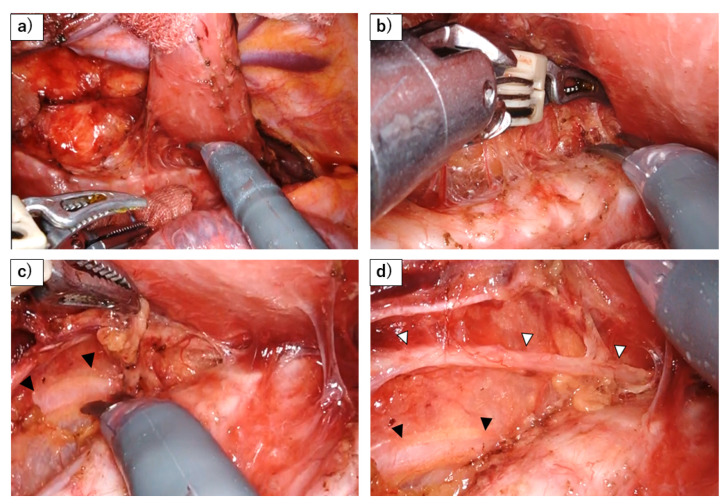
Dissection of left RLN lymph nodes using a robot. (**a**) A stable surgical field is achieved by retracting the esophagus dorsally with gauze and using the robot to fix the trachea in place. (**b**) Dissection of the left side of the trachea from the lymphatic chain. The use of the robot allows for precise hemostasis while maneuvering over the trachea. (**c**) The sympathetic cardiac branch (black arrowhead) is revealed behind the thin membrane, as the lymphatic chain is flipped up. (**d**) The left RLN (white arrowhead) and its esophageal branch have been preserved, after flipping up the lymphatic chain.

To prevent RLN palsy and ensure sufficient LN dissection around RLN, the most important consideration is to perform the dissection at the anatomically appropriate plane (Figure 1 and Figure 2). This does not mean isolating the RLN by digging this nerve out from the surrounding adipose tissue but rather dissecting the mesentery-like tissue that contains the lymph nodes around the nerve [35]. Achieving this requires not only a thorough understanding of the relevant anatomy but also adhering to fundamental surgical techniques, such as minimizing bleeding to maintain a clear view of the anatomical structures, avoiding rough manipulation around the nerve, and refraining from the use of energy devices in close proximity to the nerve. Based on those principles, recent efforts have introduced various techniques for further reducing the risk of RLN injury such as the concept of maintaining the RLN anatomical position to avoid any stress on the RLN [36], the use of mini-clips [37], magnetic anchoring [38], the and elastic suspension of the left RLN [39].

**Figure 3:**
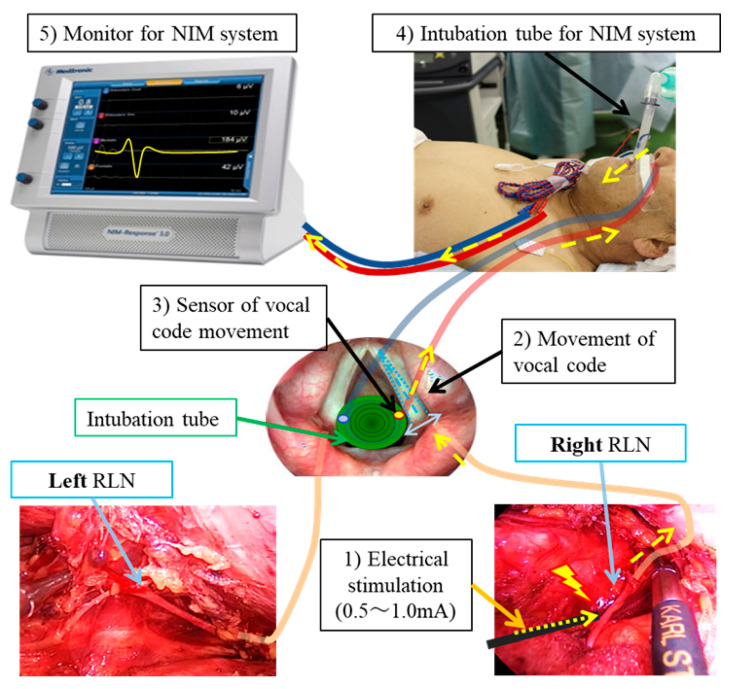
Schematic diagram of NIM (Nerve Integrity Monitoring). This diagram illustrates the mechanism of electrical stimulation of the vagus and RLN, which causes vocal cord movement detected via sensors attached to the endotracheal tube. The process is as follows: (**1**) The RLN is stimulated with a current of 0.5–1.0 mA. (**2**) The vocal cords move in response to the stimulation. (**3**) The sensor detects this vocal code movement. (**4**) The signal is transmitted as electrical impulses through the cord of the NIM endotracheal tube. (**5**) The stimulation is displayed as an electromyographic signal on the NIM monitor. Yellow arrows: Passage of electrical stimulation and signal to NIM system.

The use of a Nerve Integrity Monitor (NIM) is also an effective method for reducing the incidence of RLN injury during esophagectomy. The principle of NIM involves the placement of electrodes on the vocal cords via an endotracheal tube, which detect electromyographic (EMG) signals from the vocal code. When the RLN is stimulated by electrical impulses during surgery, the resulting EMG activity provides real-time feedback on the nerve’s functional integrity, allowing for the precise identification of RLN and avoiding inadvertent nerve damage during surgery (Figure 3). Several studies have demonstrated that the usage of NIM significantly decreases the risk of RLN palsy compared to traditional surgical methods [40,41,42,43]. Furthermore, continuous NIM provides real-time feedback on RLN function by continuously assessing electromyographic signals from the vocal cords, providing immediate feedback on RLN function throughout the surgical procedure [44,45]. This real-time feedback alerts the surgeon to any impending nerve injury, enabling immediate corrective actions, such as adjusting surgical maneuvers, thus preventing permanent RLN damage. However, there are some limitations to this continuous NIM, such as the need for specialized equipment and extra surgical time to attach the sensor to the cervical vagal nerve before approaching the RLN.

**Figure 4:**
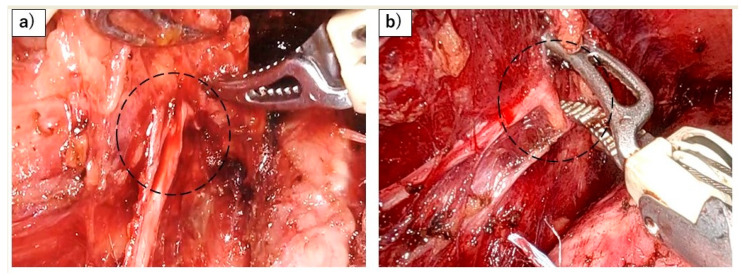
Risk of RLN palsy associated with the application of strong forces during robotic esophagectomy. (**a**) Flexion of the left RLN (black dots) caused by the forceful elevation of the esophagus using the robot. (**b**) Flexion of the left RLN (black dots) resulting from powerful robotic dissection.

Robot-assisted minimally invasive esophagectomy (RAMIE) has gained attention as a promising approach to reducing RLN palsy during esophageal cancer surgery. Using the robot, the enhanced dexterity, precision, and three-dimensional visualization offered by robotic systems are thought to facilitate meticulous dissection along the anatomical layers, potentially lowering the risk of RLN injury compared to conventional MIE [46]. Although one multicenter randomized clinical trial (RCT) demonstrates the superiority of RAMIE in preventing RLN palsy (20% vs. 34%) [47], another multicenter RCT reported differing results (33% vs. 27%) [48]. Furthermore, many meta-analyses have failed to show a definitive benefit of RAMIE [49]. One potential explanation for these discrepancies may be due the strong forces exerted by the use of the robot, which can result in more forceful retraction of the esophagus and increased flexion of the nerve, potentially contributing to RLN palsy (Figure 4). However, these issues could improve with technological advancements and enhanced operator skill. To establish the true effectiveness of RAMIE in preventing RLN palsy, prospective, multicenter trials with standardized methodologies are needed, particularly as RAMIE becomes more widely adopted.

As a new approach, the use of artificial intelligence (AI) in preventing RLN palsy during esophageal surgery has shown promising potential [50]. AI systems enhance RLN preservation by utilizing real-time image analysis and machine learning algorithms. However, there remain some issues including the need for large datasets for AI training and difficulties in integrating AI into clinical workflows.

## 5. Management

If the RLN has been severed or irreversibly damaged during surgery, reinnervation techniques are critical for restoring laryngeal function and improving patient outcomes. Although primary end-to-end anastomosis is desirable for RLN repair, usually both nerve stumps are not available. Laryngeal reinnervation using the ansa cervicalis nerve for the RLN can be performed even when the proximal RLN stump is not available, and this procedure has been shown to improve glottic closure and vocal quality and reduce aspiration by redirecting motor inputs from the ansa cervicalis to the denervated laryngeal muscles [51,52]. This nerve anastomosis is performed using 7-0 or 8-0 polypropylene and is typically carried out under a microscope by plastic surgeons or otolaryngologists—head and neck surgeons who have the skill to suture the nerve. Although this technique does not fully restore normal nerve function, it significantly enhances laryngeal competence and voice quality compared to non-reinnervated cases.

When recurrent nerve palsy occurs, its treatment differs depending on whether it is bilateral or unilateral. Patients with bilateral RLN palsy have a narrow rima glottidis, impaired airway function, and inspiratory dyspnea. This complication can be fatal, especially in postoperative esophageal cancer patients due to the high sputum production by a thoracic procedure and the longer operation time, often leading to life-threatening respiratory distress. Although about two-thirds of patients with bilateral RLN palsy could avoid tracheostomy according to previous reports [12,14,15], this tracheotomy should not be delayed in order to avoid fatal complications, as the tracheal tube can be removed once the patient’s condition improves. In cases in which bilateral RLN palsy lasted for a long time without severe complications but glottic stenosis had not improved, many treatment options were still available such as laser cordotomy, endoscopic glottic expansion, laryngeal botox injections, and selective laryngeal reinnervation [53,54,55,56]. Although these procedures may improve airway patency, consultation with an ENT specialist is essential to determine the appropriate indication.

When RLN palsy occurred unilaterally, or bilaterally but without any respiratory distress, the following management would contribute to patient recovery. Rehabilitation is a critical component in the management of RLN palsy, aiming to restore vocal function, improve swallowing, and enhance overall quality of life. Voice therapy targets exercises that strengthen the laryngeal muscles, improve glottic closure, and enhance breath support during phonation [57,58]. Swallowing rehabilitation is also crucial for patients with RLN palsy who experience dysphagia and aspiration risks. Swallowing therapy often includes exercises that strengthen the muscles, improve airway protection, and facilitate bolus clearance [59,60]. In addition to this rehabilitation, combination with neuromuscular electrical stimulation targeting the pharyngeal muscles demonstrated a certain efficacy in preventing aspiration in stroke patients [61,62]. Although further investigation is needed to establish its effectiveness for the condition of RLN palsy, this technique may hold potential for preventing aspiration in those RLN palsy patients.

Usually, RLN palsy improves within a few months, and if aspiration can be prevented during this period through rehabilitation, the problem is solved. However, if the RLN palsy does not improve and aspiration continues, surgical intervention, such as intracordal injection, thyroplasty, and arytenoid adduction, would be the next treatment option [63,64]. Intracordal injection involves injecting materials such as autologous fat, collagen, or synthetic fillers into the paralyzed vocal fold to improve glottic closure and voice quality [63,64,65,66]. This procedure is minimally invasive and can be performed under local anesthesia. Therefore, in some institutions, this injection is considered an early treatment option for RLN palsy to improve swallowing function, reduce pulmonary complications, and lower the risk of surgical intervention [67,68,69]. However, multiple intervention may be necessary to maintain the effect as the injected material can be reabsorbed over time. Before proceeding with direct surgery on the larynx, ansa-to-RLN nonselective laryngeal reinnervation is also commonly employed for chronic RLN palsy, as long as the structural integrity of the distal nerve remains intact [70]. Thyroplasty, specifically Type I thyroplasty, involves implanting a silicone or other synthetic material medially within the thyroid cartilage to reposition the paralyzed vocal fold [63,64]. This procedure is designed to provide a permanent improvement in voice quality and glottic closure. It is highly effective in patients with unilateral vocal cord palsy, as it restores vocal fold tension and improves voice projection. Arytenoid adduction is a more complex surgical approach that repositions the arytenoid cartilage to improve glottic closure by mimicking the normal position of the vocal fold during phonation [64,71]. This technique is particularly beneficial when there is a significant posterior glottic gap that cannot be adequately addressed by thyroplasty alone.

If aspiration pneumonia is repeated despite these efforts, laryngotracheal separation, specifically the Lindeman procedure [72], may be necessary to prevent aspiration by separating the larynx from the trachea. In recent years, various innovations have been added to this technique and have demonstrated improved safety profiles and reduced complications, making them valuable alternatives to the traditional Lindeman procedure [73,74]. However, speech function is permanently lost even with these technological advances. To avoid this situation, which has a significant impact on quality of life, it is necessary to place emphasis on preventing severe RLN injury during surgery.

## 6. Conclusions

RLN palsy remains a significant challenge in MIE, with varying incidence rates depending on surgical techniques and patient factors. While RLN palsy can lead to complications such as vocal cord dysfunction, dysphagia, and respiratory issues, its impact on long-term survival is uncertain. Preventive measures like NIM and RAIMIE might be useful, but further validation is needed. Management strategies, including rehabilitation and surgical interventions, aim to restore function and improve outcomes. Future research efforts should focus on refining preventive measures and optimizing therapeutic interventions to mitigate the impact of RLN palsy on patient quality of life following esophagectomy.

## References

[1] Ninomiya I., Osugi H., Fujimura T., Fushida S., Okamoto K., Maruzen S., Oyama K., Kinoshita J., Tsukada T., Kitagawa H. (2014). Thoracoscopic esophagectomy with extended lymph node dissection in the left lateral position: Technical feasibility and oncologic outcomes. Dis. Esophagus.

[2] Jang H.J., Lee H.S., Kim M.S., Lee J.M., Zo J.I. (2011). Patterns of lymph node metastasis and survival for upper esophageal squamous cell carcinoma. Ann. Thorac. Surg..

[3] Kanemura T., Makino T., Miyazaki Y., Takahashi T., Kurokawa Y., Yamasaki M., Nakajima K., Takiguchi S., Mori M., Doki Y. (2017). Distribution patterns of metastases in recurrent laryngeal nerve lymph nodes in patients with squamous cell esophageal cancer. Dis. Esophagus.

[4] Taniyama Y., Nakamura T., Mitamura A., Teshima J., Katsura K., Abe S., Nakano T., Kamei T., Miyata G., Ouchi N. (2013). A strategy for supraclavicular lymph node dissection using recurrent laryngeal nerve lymph node status in thoracic esophageal squamous cell carcinoma. Ann. Thorac. Surg..

[5] Wu J., Chen Q.X., Zhou X.M., Mao W.M., Krasna M.J. (2014). Does recurrent laryngeal nerve lymph node metastasis really affect the prognosis in node-positive patients with squamous cell carcinoma of the middle thoracic esophagus?. BMC Surg..

[6] Hong T.H., Kim H.K., Lee G., Shin S., Cho J.H., Choi Y.S., Zo J.I., Shim Y.M. (2022). Role of recurrent laryngeal nerve lymph node dissection in surgery of early-stage esophageal squamous cell carcinoma. Ann. Surg. Oncol..

[7] Xu S., Chen D., Liu Z., Song P., Zheng Y., Xue X., Sang Y., Li Z., Chen Y. (2023). Impact of the extent of recurrent laryngeal nerve lymphadenectomy on thoracic esophageal squamous cell carcinoma: A real-world multicentre study. Eur. J. Cardiothorac. Surg..

[8] Liu F., Yang W., Yang W., Xu R., Chen L., He Y., Liu Z., Zhou F., Hou B., Zhang L. (2023). Minimally invasive or open esophagectomy for treatment of resectable esophageal squamous cell carcinoma? Answer from a real-world multicenter study. Ann. Surg..

[9] Mao Y., Gao S., Li Y., Chen C., Hao A., Wang Q., Tan L., Ma J., Xiao G., Fu X. (2023). Minimally invasive versus open esophagectomy for resectable thoracic esophageal cancer (NST 1502): A multicenter prospective cohort study. J. Natl. Cancer Cent..

[10] Na K.J., Kang C.H., Park S., Park I.K., Kim Y.T. (2022). Robotic esophagectomy versus open esophagectomy in esophageal squamous cell carcinoma: A propensity-score matched analysis. J. Robot. Surg..

[11] Takeuchi H., Miyata H., Ozawa S., Udagawa H., Osugi H., Matsubara H., Konno H., Seto Y., Kitagawa Y. (2017). Comparison of short-term outcomes between open and minimally invasive esophagectomy for esophageal cancer using a nationwide database in Japan. Ann. Surg. Oncol..

[12] Taniyama Y., Miyata G., Kamei T., Nakano T., Abe S., Katsura K., Sakurai T., Teshima J., Hikage M., Ohuchi N. (2015). Complications following recurrent laryngeal nerve lymph node dissection in esophageal cancer surgery. Interact. Cardiovasc. Thorac. Surg..

[13] Li Z.G., Zhang X.B., Wen Y.W., Liu Y.H., Chao Y.K. (2018). Incidence and predictors of unsuspected recurrent laryngeal nerve lymph node metastases after neoadjuvant chemoradiotherapy in patients with esophageal squamous cell carcinoma. World J. Surg..

[14] Lee J.O., Yun J.K., Jeong Y.H., Lee Y.S., Kim Y.H. (2024). Management for recurrent laryngeal nerve paralysis following oesophagectomy for oesophageal cancer: Thoracic surgeon perspective. J. Thorac. Dis..

[15] Jeon Y.J., Cho J.H., Lee H.K., Kim H.K., Choi Y.S., Zo J.I., Shim Y.M. (2021). Management of patients with bilateral recurrent laryngeal nerve paralysis following esophagectomy. Thorac. Cancer.

[16] Van Workum F., Berkelmans G.H., Klarenbeek B.R., Nieuwenhuijzen G.A.P., Luyer M.D.P., Rosman C. (2017). McKeown or Ivor Lewis totally minimally invasive esophagectomy for cancer of the esophagus and gastroesophageal junction: Systematic review and meta-analysis. J. Thorac. Dis..

[17] Wang J., Hu J., Zhu D., Wang K., Gao C., Shan T., Yang Y. (2020). McKeown or Ivor Lewis minimally invasive esophagectomy: A systematic review and meta-analysis. Transl. Cancer Res..

[18] Saito Y., Takeuchi H., Fukuda K., Suda K., Nakamura R., Wada N., Kawakubo H., Kitagawa Y. (2018). Size of recurrent laryngeal nerve as a new risk factor for postoperative vocal cord paralysis. Dis. Esophagus.

[19] Ohi M., Toiyama Y., Yasuda H., Ichikawa T., Imaoka H., Okugawa Y., Fujikawa H., Okita Y., Yokoe T., Hiro J. (2021). Preoperative computed tomography predicts the risk of recurrent laryngeal nerve paralysis in patients with esophageal cancer undergoing thoracoscopic esophagectomy in the prone position. Esophagus.

[20] Liu N., Chen B., Li L., Zeng Q., Sheng L., Zhang B., Liang W., Lv B. (2020). Mechanisms of recurrent laryngeal nerve injury near the nerve entry point during thyroid surgery: A retrospective cohort study. Int. J. Surg..

[21] Hayami M., Watanabe M., Mine S., Imamura Y., Okamura A., Yuda M., Yamashita K., Shoji Y., Toihata T., Kozuki R. (2020). Steam induced by the activation of energy devices under a wet condition may cause thermal injury. Surg. Endosc..

[22] Sulica L. (2008). The natural history of idiopathic unilateral vocal fold paralysis: Evidence and problems. Laryngoscope.

[23] Lee D.H., Lee S.Y., Lee M., Seok J., Park S.J., Jin Y.J., Lee D.Y., Kwon T.K. (2020). Natural course of unilateral vocal fold paralysis and optimal timing of permanent treatment. JAMA Otolaryngol. Head Neck Surg..

[24] Woodson G. (2021). Pathophysiology of recurrent laryngeal nerve injury. Surgery of the Thyroid and Parathyroid Glands.

[25] Kuo C.T., Chiu C.H., Fang T.J., Chao Y.K. (2024). Prognostic factors for recovery from left recurrent laryngeal nerve palsy after minimally invasive McKeown esophagectomy: A retrospective study. Ann. Surg. Oncol..

[26] Shimizu H., Shiozaki A., Fujiwara H., Konishi H., Kosuga T., Komatsu S., Ichikawa D., Okamoto K., Otsuji E. (2017). Short- and long-term progress of recurrent laryngeal nerve paralysis after subtotal esophagectomy. Anticancer Res..

[27] Takatsu J., Higaki E., Abe T., Fujieda H., Yoshida M., Yamamoto M., Shimizu Y. (2024). Critical swallowing functions contributing to dysphagia in patients with recurrent laryngeal nerve paralysis after esophagectomy. Esophagus.

[28] Baba M., Aikou T., Natsugoe S., Kusano C., Shimada M., Nakano S., Fukumoto T., Yoshinaka H. (1998). Quality of life following esophagectomy with three-field lymphadenectomy for carcinoma, focusing on its relationship to vocal cord palsy. Dis. Esophagus.

[29] Scholtemeijer M.G., Seesing M.F.J., Brenkman H.J.F., Janssen L.M., van Hillegersberg R., Ruurda J.P. (2017). Recurrent laryngeal nerve injury after esophagectomy for esophageal cancer: Incidence, management, and impact on short- and long-term outcomes. J. Thorac. Dis..

[30] Oshikiri T., Takiguchi G., Hasegawa H., Yamamoto M., Kanaji S., Yamashita K., Matsuda T., Nakamura T., Suzuki S., Kakeji Y. (2021). Postoperative recurrent laryngeal nerve palsy is associated with pneumonia in minimally invasive esophagectomy for esophageal cancer. Surg. Endosc..

[31] Yang Y., Li B., Xu X., Liu Z., Jiang C., Wu X., Yang Y., Li Z. (2023). Short-term and long-term effects of recurrent laryngeal nerve injury after robotic esophagectomy. Eur. J. Surg. Oncol..

[32] Booka E., Takeuchi H., Nishi T., Matsuda S., Kaburagi T., Fukuda K., Nakamura R., Takahashi T., Wada N., Kawakubo H. (2015). The impact of postoperative complications on survival after esophagectomy for esophageal cancer. Medicine.

[33] Qu R., Tu D., Ping W., Fu X. (2021). The impact of recurrent laryngeal nerve injury on prognosis after McKeown esophagectomy for ESCC. Cancer Manag. Res..

[34] Kelly R.J., Ajani J.A., Kuzdzal J., Zander T., Van Cutsem E., Piessen G., Mendez G., Feliciano J., Motoyama S., Lièvre A. (2021). Adjuvant Nivolumab in Resected Esophageal or Gastroesophageal Junction Cancer. N. Engl. J. Med..

[35] Tsunoda S., Shinohara H., Kanaya S., Okabe H., Tanaka E., Obama K., Hosogi H., Hisamori S., Sakai Y. (2020). Mesenteric excision of upper esophagus: A concept for rational anatomical lymphadenectomy of the recurrent laryngeal nodes in thoracoscopic esophagectomy. Surg. Endosc..

[36] Otsuka K., Murakami M., Goto S., Ariyoshi T., Yamashita T., Saito A., Kohmoto M., Kato R., Lefor A.K., Aoki T. (2020). Minimally invasive esophagectomy and radical lymph node dissection without recurrent laryngeal nerve paralysis. Surg. Endosc..

[37] Saeki H., Nakashima Y., Hirose K., Sasaki S., Jogo T., Taniguchi D., Edahiro K., Korehisa S., Kudou K., Nakanishi R. (2018). “Energy-less technique” with mini-clips for recurrent laryngeal nerve lymph node dissection in prone thoracoscopic esophagectomy for esophageal cancer. Am. J. Surg..

[38] Fu J., Li Y., Wang Z., Cheng Y., Chen N., Sun X., Zhang B., Peng Z., Chen W., Qian R. (2022). The role of magnetic anchoring and traction technique in thoracoscopic lymphadenectomy along the left recurrent laryngeal nerve. Surg. Endosc..

[39] Dongming G., Yuequan J., Qi Z., Huajie X., Zhiqiang W. (2023). A novel technique for lymphadenectomy along the left recurrent laryngeal nerve during minimally invasive esophagectomy: A retrospective cohort study. BMC Surg..

[40] Hikage M., Kamei T., Nakano T., Abe S., Katsura K., Taniyama Y., Sakurai T., Teshima J., Ito S., Niizuma N. (2017). Impact of routine recurrent laryngeal nerve monitoring in prone esophagectomy with mediastinal lymph node dissection. Surg. Endosc..

[41] Kanemura T., Miyata H., Yamasaki M., Makino T., Miyazaki Y., Takahashi T., Kurokawa Y., Takiguchi S., Mori M., Doki Y. (2019). Usefulness of intraoperative nerve monitoring in esophageal cancer surgery in predicting recurrent laryngeal nerve palsy and its severity. Gen. Thorac. Cardiovasc. Surg..

[42] Huang C.L., Chen C.M., Hung W.H., Cheng Y.F., Hong R.P., Wang B.Y., Cheng C.Y. (2022). Clinical outcome of intraoperative recurrent laryngeal nerve monitoring during thoracoscopic esophagectomy and mediastinal lymph node dissection for esophageal cancer. J. Clin. Med..

[43] Chen B., Yang T., Wang W., Tang W., Xie J., Kang M. (2023). Application of intraoperative neuromonitoring (IONM) of the recurrent laryngeal nerve during esophagectomy: A systematic review and meta-analysis. J. Clin. Med..

[44] Wong I., Tong D.K.H., Tsang R.K.Y., Wong C.L.Y., Chan D.K.K., Chan F.S.Y., Law S. (2017). Continuous intraoperative vagus nerve stimulation for monitoring of recurrent laryngeal nerve during minimally invasive esophagectomy. J. Vis. Surg..

[45] Komatsu S., Konishi T., Matsubara D., Soga K., Shimomura K., Ikeda J., Taniguchi F., Fujiwara H., Shiozaki A., Otsuji E. (2022). Continuous recurrent laryngeal nerve monitoring during single-port mediastinoscopic radical esophagectomy for esophageal cancer. J. Gastrointest. Surg..

[46] Oshikiri T., Goto H., Horikawa M., Urakawa N., Hasegawa H., Kanaji S., Yamashita K., Matsuda T., Nakamura T., Kakeji Y. (2021). Incidence of recurrent laryngeal nerve palsy in robot-assisted versus conventional minimally invasive McKeown esophagectomy in prone position: A propensity score-matched study. Ann. Surg. Oncol..

[47] Chao Y.K., Li Z., Jiang H., Wen Y.W., Chiu C.H., Li B., Shang X., Fang T.J., Yang Y., Yue J. (2024). Multicentre randomized clinical trial on robot-assisted versus video-assisted thoracoscopic oesophagectomy (REVATE trial). Br. J. Surg..

[48] Yang Y., Li B., Yi J., Hua R., Chen H., Tan L., Li H., He Y., Guo X., Sun Y. (2022). Robot-assisted versus conventional minimally invasive esophagectomy for resectable esophageal squamous cell carcinoma: Early results of a multicenter randomized controlled trial: The RAMIE trial. Ann. Surg..

[49] Watanabe M., Kuriyama K., Terayama M., Okamura A., Kanamori J., Imamura Y. (2023). Robotic- Assisted Esophagectomy: Current Situation and Future Perspectives. Ann. Thorac. Cardiovasc. Surg..

[50] Sato K., Fujita T., Matsuzaki H., Takeshita N., Fujiwara H., Mitsunaga S., Kojima T., Mori K., Daiko H. (2022). Real-time detection of the recurrent laryngeal nerve in thoracoscopic esophagectomy using artificial intelligence. Surg. Endosc..

[51] Natsugoe S., Okumura H., Matsumoto M., Ishigami S., Owaki T., Nakano S., Aikou T. (2005). Reconstruction of recurrent laryngeal nerve with involvement by metastatic node in esophageal cancer. Ann. Thorac. Surg..

[52] Koyanagi K., Igaki H., Iwabu J., Ochiai H., Tachimori Y. (2015). Recurrent laryngeal nerve paralysis after esophagectomy: Respiratory complications and role of nerve reconstruction. Tohoku J. Exp. Med..

[53] Filauro M., Vallin A., Marcenaro E., Missale F., Fragale M., Mora F., Marrosu V., Sampieri C., Carta F., Puxeddu R. (2021). Quality of life after transoral CO_2_ laser posterior cordotomy with or without partial arytenoidectomy for bilateral adductor vocal cord paralysis. Eur. Arch. Otorhinolaryngol..

[54] de Almeida R.B.S., Costa C.C., Lamounier E., Silva Duarte P., Rocha A.K.P.B., Bernardes M.N.D., Garcia J.L., Freitas L.B., Ramos H.V.L. (2023). Surgical treatment applied to bilateral vocal fold paralysis in adults: Systematic review. J. Voice.

[55] Lewis S., Woo P. (2018). Botulinum toxin in management of synkinesis in patients with unilateral and bilateral vocal fold paralysis. Laryngoscope.

[56] Marina M.B., Marie J.P., Birchall M.A. (2011). Laryngeal reinnervation for bilateral vocal fold paralysis. Curr. Opin. Otolaryngol. Head Neck Surg..

[57] Mattioli F., Menichetti M., Bergamini G., Molteni G., Alberici M.P., Luppi M.P., Nizzoli F., Presutti L. (2015). Results of early versus intermediate or delayed voice therapy in patients with unilateral vocal fold paralysis: Our experience in 171 patients. J. Voice.

[58] Walton C., Carding P., Flanagan K. (2018). Perspectives on voice treatment for unilateral vocal fold paralysis. Curr. Opin. Otolaryngol. Head Neck Surg..

[59] Kojima K., Fukushima T., Kurita D., Matsuoka A., Ishiyama K., Oguma J., Daiko H. (2023). Perioperative decrease in tongue pressure is an intervenable predictor of aspiration after esophagectomy. Dysphagia.

[60] Yu Y., Li Y., Lu Y., Hua X., Ma H., Li H., Wei X., Zhang J., Chen X., Liu Q. (2019). Chin-down-plus-larynx-tightening maneuver improves choking cough after esophageal cancer surgery. Ann. Transl. Med..

[61] Tarihci Cakmak E., Sen E.I., Doruk C., Sen C., Sezikli S., Yaliman A. (2023). The Effects of Neuromuscular Electrical Stimulation on Swallowing Functions in Post-stroke Dysphagia: A Randomized Controlled Trial. Dysphagia.

[62] Wang Z., Xiao Z., Shen Q., Zhao N., Zhang W. (2024). Neuromuscular Electrical Stimulation for Post-Stroke Dysphagia Treatment: A Systemic Evaluation and Meta-Analysis of Randomized Controlled Trials. Dysphagia..

[63] Chen X., Wan P., Yu Y., Li M., Xu Y., Huang P., Huang Z. (2014). Types and timing of therapy for vocal fold paresis/paralysis after thyroidectomy: A systematic review and meta-analysis. J. Voice.

[64] Hasukawa A., Mochizuki R., Sakamoto H., Shibano A., Kitahara T. (2023). Type I thyroplasty or fat injection laryngoplasty versus arytenoid adduction: Effects of surgery on voice recovery in patients with unilateral vocal fold paralysis. Ear Nose Throat J..

[65] Jeong G.E., Lee D.H., Lee Y.S., Ahn D.S., Lee D.K., Choi S.H., Nam S.Y., Kim S.Y. (2022). Treatment efficacy of voice therapy following injection laryngoplasty for unilateral vocal fold paralysis. J. Voice.

[66] Haddad R., Ismail S., Khalaf M.G., Matar N. (2022). Lipoinjection for unilateral vocal fold paralysis treatment: A systematic review and meta-analysis. Laryngoscope.

[67] Abu-Ghanem S., Rudy S., Deane S., Tsai S.W., Shih L.C., Damrose E.J., Sung C.K. (2020). Early injection laryngoplasty after surgery: 30 cases and Proposed aspiration assessment protocol. J. Voice.

[68] Chen D.W., Price M.D., LeMaire S.A., Coselli J.S., Liou N.E., Ongkasuwan J. (2018). Early versus late inpatient awake transcervical injection laryngoplasty after thoracic aortic repair. Laryngoscope.

[69] Vila P.M., Bhatt N.K., Paniello R.C. (2018). Early-injection laryngoplasty may lower risk of thyroplasty: A systematic review and meta-analysis. Laryngoscope.

[70] Torrecillas V.F., Hoffman M.R., Schiffer B., Keefe K., Smith M.E. (2024). Long-term outcomes and revision rates in laryngeal reinnervation. Laryngoscope.

[71] Watanabe K., Hirano A., Kobayashi Y., Sato T., Honkura Y., Katori Y. (2023). Long-term voice evaluation after arytenoid adduction surgery in patients with unilateral vocal fold paralysis. Eur. Arch. Otorhinolaryngol..

[72] Lindeman R.C. (1975). Diverting the paralyzed larynx: A reversible procedure for intractable aspiration. Laryngoscope.

[73] Ninomiya H., Yasuoka Y., Inoue Y., Toyoda M., Takahashi K., Miyashita M., Furuya N. (2008). Simple and new surgical procedure for laryngotracheal separation in pediatrics. Laryngoscope.

[74] Shino M., Yasuoka Y., Murata T., Ninomiya H., Takayasu Y., Takahashi K., Chikamatsu K. (2013). Improvement of tracheal flap method for laryngotracheal separation. Laryngoscope.

